# A dataset of pairs of an image and tags for cataloging image-based archives

**DOI:** 10.1016/j.dib.2022.108722

**Published:** 2022-11-04

**Authors:** Tokinori Suzuki, Kota Nagamizo, Daisuke Ikeda

**Affiliations:** Kyushu University, Fukuoka 8190395, Japan

**Keywords:** Disambiguation of image tags, Image data, Catalogue, Metadata, Wikipedia

## Abstract

The dataset described in this paper contains pairs of images collected from the Web and their tags of keywords, which are linked to appropriate entity pages of Wikipedia, and programs to reproduce experiments. It is assumed for evaluating the disambiguation task, in which given an image and its tags to be disambiguated, an appropriate Wikipedia page is selected for each of the given tag. We collected images tagged keywords of animal names for that ambiguity and their tags since animal names may refer to not only names of animal but names of other types of objects, e.g., nicknames of sports teams from the photo sharing site Flickr. The tags are linked to the correspondence Wikipedia page judged by annotators. The dataset includes 420 images and 2,464 tags. It is useful for developing a system to link a keyword of an image to an entry of a knowledgebase as well as an image classification system, which include fine-grained classes, e.g. proper nouns of objects, as their classification targets.

## Specifications Table

**Table:** 

Subject	Library and Information Sciences
Specific subject area	Archive management system, Digital library, Catalogue
Type of data	Image, Table (annotation)
How the data were acquired	Data were created by collecting pairs of an image and its assigned tags from a photo sharing site Flickr, and annotations. A program to download the image data is included in the dataset. The annotation for a tag is the corresponding Wikipedia article since keywords of the tag assigned to an image is not unique. For example, a tag ‘mouse’ stands for not only a mammal but a computer device. The English version of Wikipedia dump data as of 1st October 2017 is used to obtain Wikipedia articles.
Data format	Raw and analyzed
Data source location	Kyushu University, Fukuoka, Japan
Data accessibility	Repository name: Mendeley Data Data identification number: 10.17632/msyc6mzvhg.1 Direct URL to data: https://data.mendeley.com/datasets/msyc6mzvhg/1 License: CC BY 4.0 license

## Value of the Data

• This dataset is the first dataset for disambiguation of tags assigned to images to the best of our knowledge. It is useful for training a disambiguation system of keywords assigned to an image and evaluating the system.
• The dataset maintains correspondences between an image and a Wikipedia page. The correspondences often include granular entities, such as a Wikipedia page of a specific model of cars, that of a particular place. It is also useful to evaluate the fine-grained image classification task including granular classification labels.
• The dataset offers not only data themselves but also programs as baseline methods of the disambiguation task. It is beneficial for researchers to reproduce experiments as well as to start development of their new methods with ease.

## 1 Data Description

**Fig. 1:**
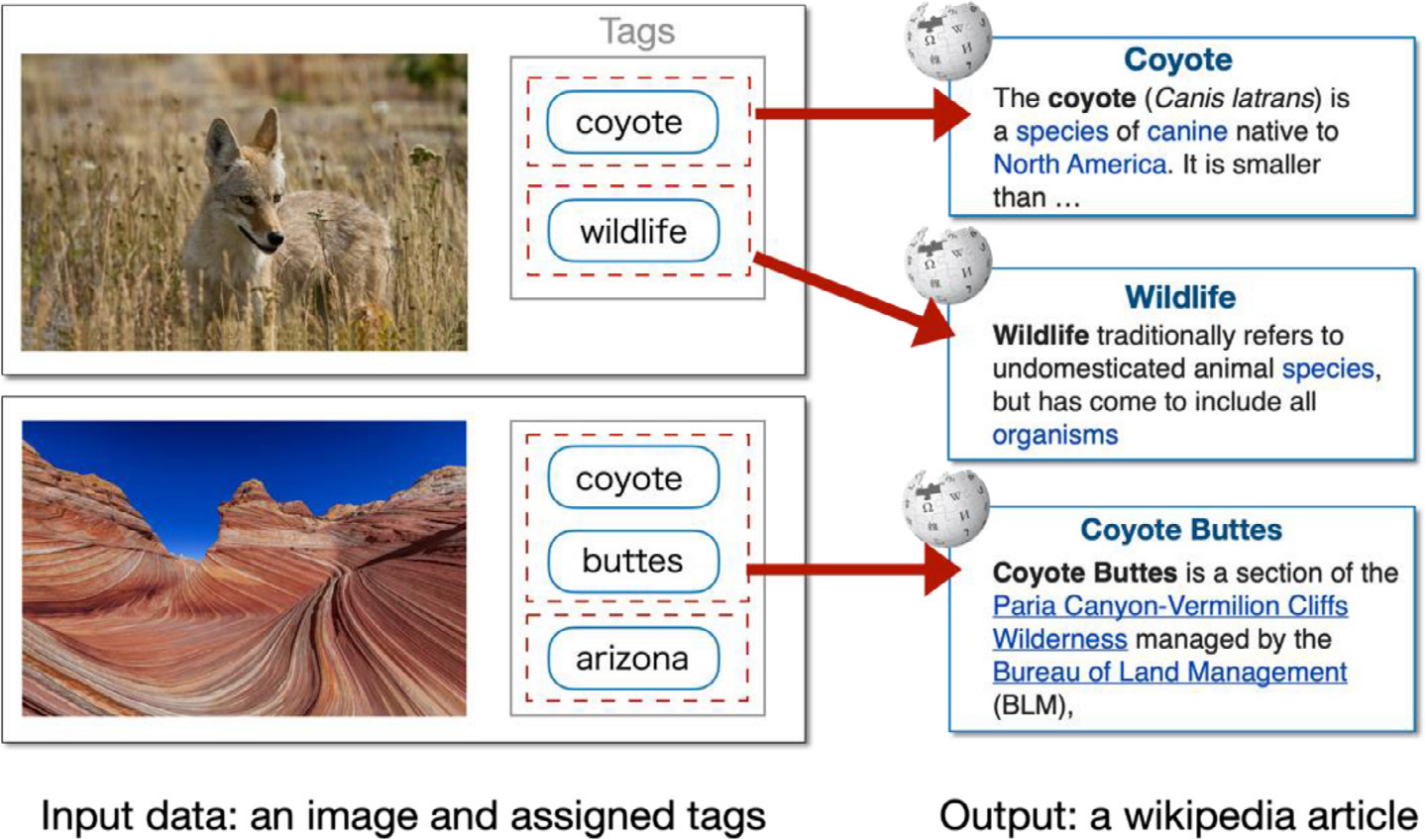
Examples of inputs and outputs of the disambiguation task.

This dataset presented in this paper is developed to evaluate the disambiguation task proposed in [6], in which an image and its associated tags are provided as a query, a system in response identifies an entry represented by tags in Wikipedia. The dataset consists of images tagged keywords of animal names for that ambiguity and their tags since animal names may refer to not only names of animal but names of other types of objects, e.g., nicknames of sports teams. Fig. 1 illustrates the overview of the task. The two example pictures have the same tag ‘coyote’ which is used to refer to different types of entities. The picture on the above is that of the wild dog called coyote whereas the one on the below is that of buttes located on the middle of Kanab, Utah and Page, Arizona in the U.S. The tags in the dataset often refer to granular entities, such as a particular place ‘Coyote Buttes’. Thus, the dataset can be used not only for model training and system evaluation of the tag disambiguation task but also for the fine-grained image classification, in which more specific tags are its classification target.

**Fig. 2:**
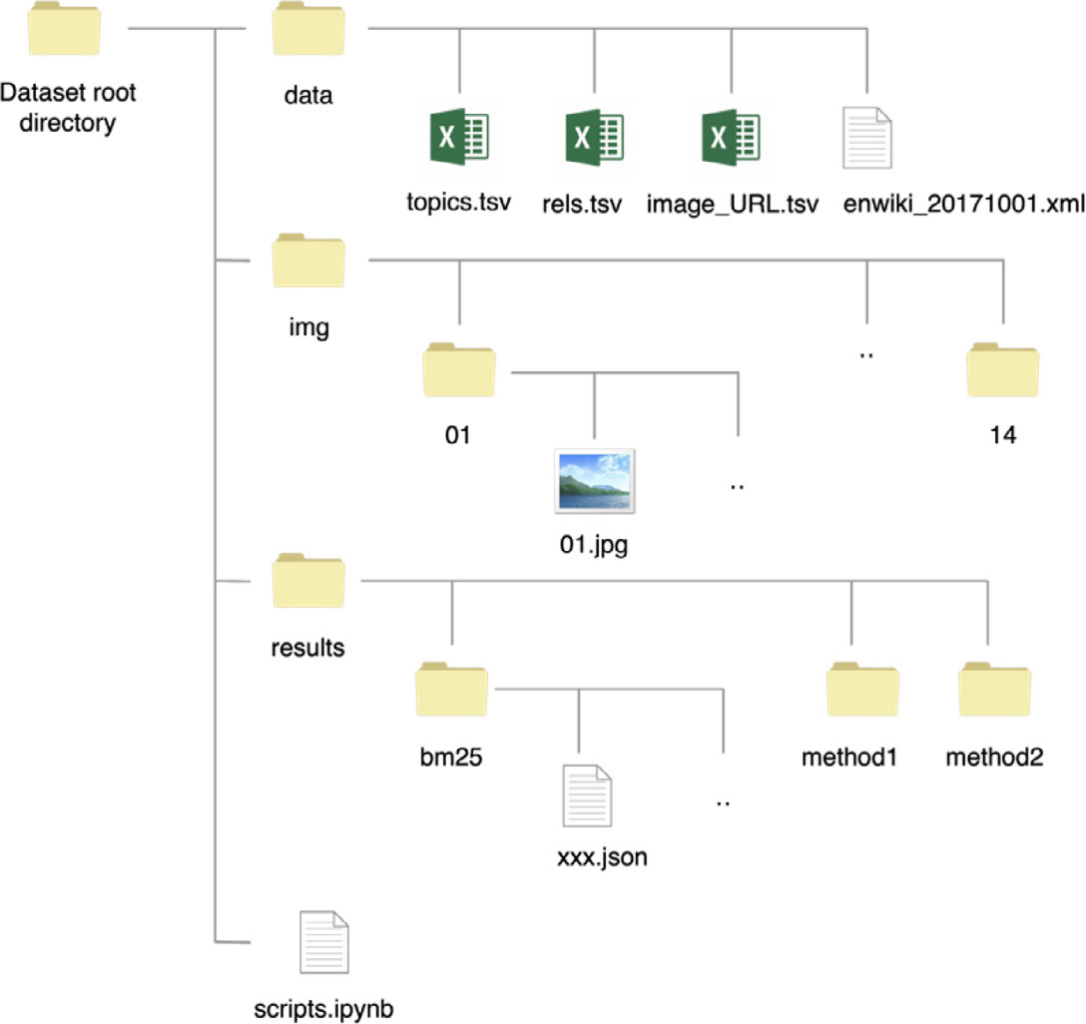
Structure of the dataset.

The dataset contains 420 images and 2,464 tags linked with their associated Wikipedia articles. The images and tags are collected from Flickr^1^, and the Wikipedia articles for each tag are judged by annotators. The dataset is mainly organized in three directories and one script file as depicted in Fig. 2. Each of the components are introduced in the following sections.

### 1.1 Data directory

The data directory contains three tables and an XML file. The three tables, topics.txt, rels.txt and image_URL.txt are tab-separated files. First, topics.txt contains a set of a disambiguation tags, i.e. query tag and its associated image and other tags, which we call a topic. Table 1 shows the fields of the topic table and an example record. Second, rels.txt contains answers of correct Wikipedia pages for each topic. The fields of the file are shown in Table 2. Last, image_URL.txt lists the original Flickr URLs of image files. Table 3 shows the fields of the file. A download program of images is arranged in scripts.ipynb using the URL file.

**Table 1:** Structure of the table in topics.txt.

Field name	Data description	Data format	Example
Topic	Identifier of topic	Integer number	010102
Title	Title of topic	String	Albatross_01-2
Query_tag	A target tag to be disambiguated	String	albatross
All_tags	All of the tags assigned to an image	List of strings separated by comma	wanderingalbatross,albatross
Image_file	File name of an image linked to the topic	String	01-01.img

**Table 2:** Structure of the table in rels.txt.

Field name	Data description	Data format	Example
Topic	Identifier of a topic	Integer number	010102
Title	Correct title of article for the topic	String	Albatross
URL	Wikipedia URL of the correct article	String	https://en.wikipedia.org/wiki/Albatross

**Table 3:** Structure of the table in image_URL.txt.

Field name	Data description	Data format	Example
Image_file	File name of an image	string	01-02.jpg
URL	URL of the image file	String	https://www.flickr.com/photos/12413896@N03/2483330091

**Fig. 3:**
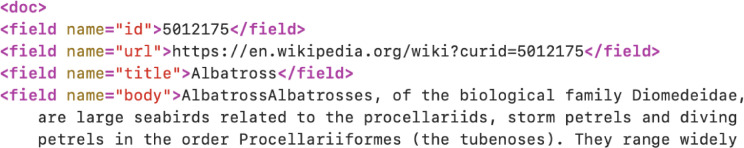
An example of Wikipedia article in enwiki_20171001.xml.

An XML file ‘enwiki_20171001.xml’ contains Wikipedia 1,210,453 articles of English version. The article information was extracted from Wikipedia dump data as of 1st October 2017^2^ with WikiExtractor^3^, a python library. The elements of individual article of the file are id, URL, title, and the body of the article. An example of the article of the albatross in the file is displayed in Fig. 3. In the example, the id ‘5012175’ and the URL ‘https://en.wikipedia.org/wiki?curid=5012175’ are derived from Wikipedia identifier. The text of the albatross is stored in the content of the body tag.

### 1.2 Image directory

The img directory is a placeholder directory in which image files (.jpg) are downloaded after executing a download program in scripts.ipynb. The directory contains 420 jpg files in total. The directory structure under img is formatted in ‘xx/yy.jpg’ where xx is a sub-directory for identifier of one of the categories, i.e. names of animals, yy is an identifier of pictures in the category.

### 1.3 Results directory

The results directory contains output results files of baseline systems for the disambiguation task. Under the directory, there are sub-directories linked with individual baseline systems, such as ‘bm25’ shown in Fig. 2. The result file of each topic called ‘bm25/xxxxx.json’, where xxxxx stands for Topic ID in Table 1, contains candidate Wikipedia pages for the topic.

### 1.4 Script file

**Fig. 4:**
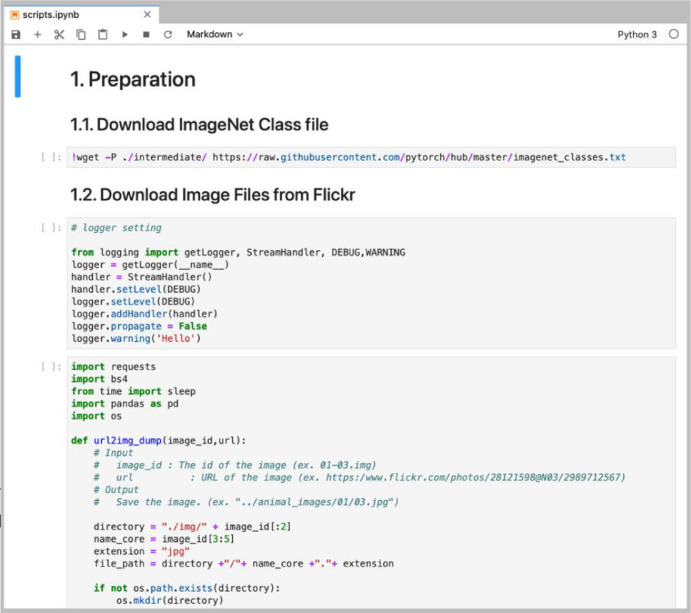
Screenshot of Jupyter notebook.

Programs to reproduce experiments for the disambiguation task are noted in a Jupyter notebook^4^ file scripts.ipynb. The file carries Python scripts of downloading image files, conducting some baseline methods for the task and evaluating the results. Fig. 4 shows a screenshot of the notebook. Each of the scripts is displayed in a cell and executable in the Jupyter notebook environment.

## 2 Experimental Design, Materials and Methods

This dataset was created by collecting images and their associated meta data and annotation. In this section, we describe the procedures of the collection and annotation.

### 2.1 Collecting images and associated tags

The images in this dataset were downloaded from Flickr. The images are assigned one of our defined 14 keywords of animals. All of the animal names are listed in the x-axis of Fig. 6. The keywords of animal name are randomly selected from a Wikipedia page of general animal names^5^ as of 1st September 2017. Because names of animals are often ambiguous, e.g. they are often used as brand names of products or nicknames of sports teams, the collected images are not limited to pictures of animals, but included any genre, e.g., automobile and airplane. all the tags assigned to the downloaded images were also collected. Tags displaying camera specifications, e.g. ‘nikond5’ and ‘500mmf4’, had been eliminated.

This dataset provide access to the images with offering the source URLs and a downloading script due to a copyright issue, similar to the way used in a famous dataset for image data, ImageNet [2]. The URL of the images are available in image_URL.txt, and a script for downloading all the images file is also available in script.ipynb.

### 2.2 Annotation

**Fig. 5:**
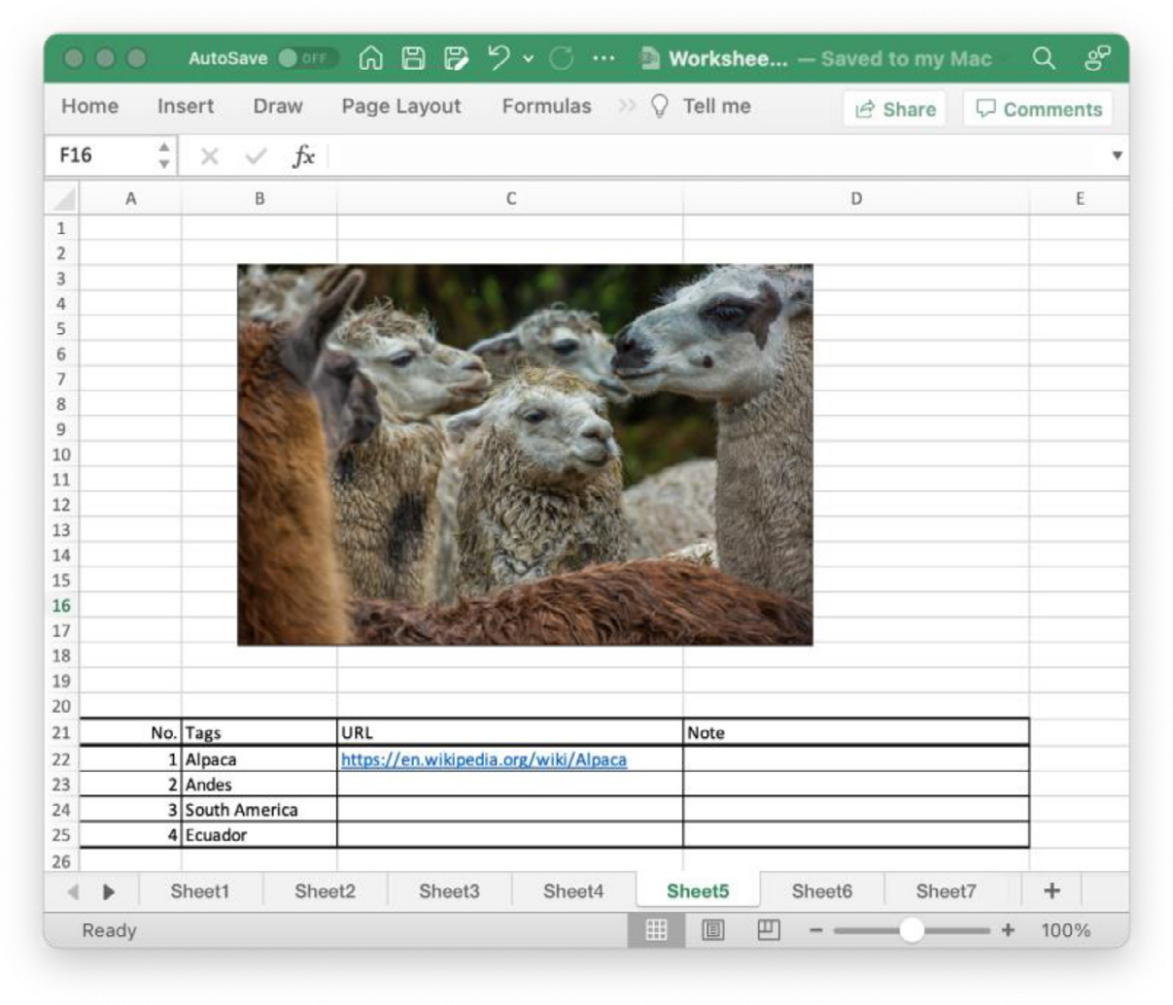
Screenshot of an annotation worksheet.

The correct Wikipedia pages paired to with each topic in rels.txt were created by annotation. In the annotation, evaluators judged correct Wikipedia pages for each tag by searching pages in Wikipedia. The annotations were conducted by following procedures:

The annotators subjectively determined correct Wikipedia page to link to tags of an image. An image and its tags were given to the annotators on a spreadsheet displayed in Fig. 5. They identified a tag (or multiple tags) which should refer to a given entity in the English version of Wikipedia regardless of whether it was depicted in the picture.

For example, using the tags ‘South’ and ‘America’ in Fig. 5, the annotators were asked to judge which Wikipedia page is corresponded to tags based on the visual contents of the given image and its other tags. Then, they searched Wikipedia pages for entities of the tags by querying their single or multiple keywords using a web browser. In this step, they were able to modify their query within their interactions with the search results of Wikipedia to obtain candidates. Lastly, they judged whether the retrieved pages were appropriate for the queried tag. If so, they wrote down the URL of the retrieved Wikipedia page in a field of URL as in Fig. 5. If they determined that the tags did not correspond to any Wikipedia page, they assigned a nil entity to the relevant tags. After three trials of the above procedures, they began annotation.

**Fig. 6:**
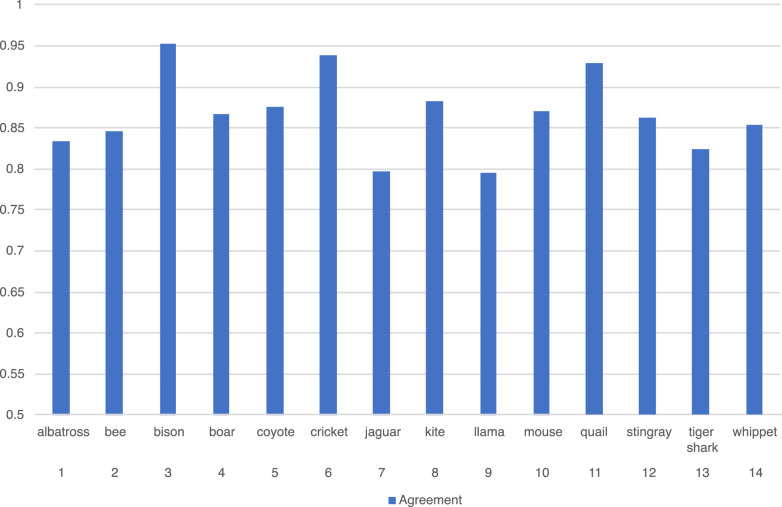
The inter-annotator agreement for each category of the dataset.

The outcomes of annotation were evaluated both on inter-annotator agreement and Cohen's κ coefficient [1]. Fig. 6 shows the inter-annotators agreement among two annotators on each category. The average of the agreement is 0.87 over the categories. The κ is above 0.9.

### 2.3 Baseline method

The dataset contains three implementations of methods, bm25, method1 and method2, as the baselines for the disambiguation task in scripts.ipynb. The results of three methods are stored in the corresponding directory of the dataset, e.g. ‘results/bm25’.

The bm25 method uses keyword search. This method retrieves Wikipedia pages querying with tags with similarity scoring of BM25 [5]. To implement BM25 ranking method, we used Apache Solr search engine^6^. The results of the bm25 method are obtained on the free parameter setting of $k_{1}=1.2,\;b=0.75$ for BM25 scoring.

The rest of the two methods, method1 and method2 are reranking methods based on the results of the bm25 method. Given the retrieval results of the bm25 as candidates for a queried tags, the two methods refine the ranking of the candidates. The method1 employs contextual information from all the tags using *word2vec* [4] to refine the candidates. The method2 employs both contextual information and the Alexnet image classifier [3]. The image classifier is a pre-rained on 1,000 object classes^7^. Replacing the output layer of the model, the classifier output one of the 14 animal names of the dataset in response to an input image.

Given the candidate Wikipedia pages T for queried tags Q and objective labels L output by image classification, the overall similarity of method1 and 2 is computed by the following similarity function.

$$\text{sim}\left(T,\;Q,L\right)=\alpha \frac{1}{\left|T\right|}\sum_{w_{t}\;\in \;T}\sum_{w_{q}\;\in \;Q}\text{sim}\left(w_{t},\;w_{q}\right)+\left(1-\alpha \right)\frac{1}{\left|T\right|}\sum_{w_{t}\;\in \;T}\sum_{w_{l}\;\in \;L}\text{sim}\left(w_{t},\;w_{l}\right),$$

where $w_{t},w_{q},w_{l}\;$represent a word in the titles of the candidate Wikipedia pages, a word in the tags and a word of image classification label for the queried image, respectively. The α represents a weighting parameter of similarities on tags and image classification labels. Since method1 only uses information of tags, it computes the similarity using only the left side of the function, i.e. α=1. The results of method2 in ‘results/method2’ directory are outputted under the parameter setting of α=0.5.

The similarity of two words is computed by the cosine distance of their vector representations:

$$\text{sim}\left(w_{1},\;w_{2}\right)=\cos\left(w_{1},\;w_{2}\right)\frac{w_{1}^{T}\;w_{2}}{∥w_{1}∥∥w_{2}∥},$$

This is the inner product of two word embedding vectors w of words w learned by word2vec.

### 2.4 Evaluation metrics

The dataset contains a script to evaluate a disambiguation method by three evaluation metrics, the mean reciprocal rank (MRR), the precision at first (P@1) and the precision at tenth (R@10). The script calculates the three metrics only for the topics where bm25 could retrieve the correct page in the output. MRR is calculated as follows:

$$\text{MRR}=\;\frac{1}{\left|Q\right|}\sum_{q\;\in Q}^{Q}\frac{1}{\text{ran}k_{q}},$$

where Q is a set of tags q of an image, and $\text{ran}k_{q}$ stands for the rank of the correct Wikipedia page for q in the output ranked list. The MRR becomes closer to 1 as the correct page is placed nearer the top of the list. The P@1 and P@10 is the average number of topics where the correct Wikipedia page is retrieved within first or tenth in the ranked list, respectively.

## CRediT authorship contribution statement

**Tokinori Suzuki:** Conceptualization, Methodology, Writing; **Kota Nagamizo:** Dataset preparation, Software and Programs, Validation; **Daisuke Ikeda:** Supervision, Writing, Reviewing and Editing.

## Ethics Statements

This dataset contains URL links of Flickr photos. The links are collected using the API provided by Flickr. In Flickr terms of service, hyperlinks to the site (the URL links in this dataset) are allowed to be created. The terms of service prohibit any use of distribute, resale, commercial use of photos reached using the links in this dataset. The copyrights of the photos are owned by users of Flickr. Because this dataset does not contain personal information of Flickr users, it is not necessary to anonymize the data.

## Declaration of Competing Interest

The authors declare that they have no known competing financial interests or personal relationships that could have appeared to influence the work reported in this paper.

## Data Availability

Dataset of Pairs of an Image and Tags for Cataloging Image-based Records (Original data) (Mendeley Data).

## Appendix Supplementary materials

Image, application 1

## Footnotes

1 https://www.flickr.com/

2 https://archive.org/download/enwiki-20171001/

3 https://github.com/attardi/wikiextractor

4 https://jupyter.org/

5 https://en.wikipedia.org/wiki/List_of_animal_names

6 https://lucene.apache.org/solr/

7 https://image-net.org/challenges/LSVRC/2013/browse-synsets.php
