# Supplementary material for: A dataset of pairs of an image and tags for cataloging image-based archives
Source: Data Brief. 2022 Nov 4;45:108722. doi: 10.1016/j.dib.2022.108722 (PMC9679671; doi:10.1016/j.dib.2022.108722)
Supplement: Supplementary file 1 [file mmc1.pdf]

Towards Automatic Cataloging of Image and Textual Collections with Wikipedia

Tokinori Suzuki[1[0000-0002-4715-6198]], Daisuke Ikeda[1], Petra Galuščáková[2[0000-0001-6328-7131]] and Douglas Oard[2]

[1] Kyushu University, Fukuoka 8190395, Japan
[2] University of Maryland, College Park MD 20742, USA
suzuki.tokinori.070@s.kyushu-u.ac.jp, daisuke@inf.kyushu-u.ac.jp
petra@umd.edu, oard@umd.edu

Abstract. In recent years, a large amount of multimedia data consisting of images and text have been generated in libraries through the digitization of physical materials into data for their preservation. When they are archived, appropriate cataloging metadata are assigned to them by librarians. Automatic annotations are helpful for reducing the cost of manual annotations. To this end, we propose a mapping system that links images and the associated text to entries on Wikipedia as a replacement for annotation by targeting images and associated text from photo-sharing sites. The uploaded images are accompanied by descriptive labels of contents of the sites that can be indexed for the catalogue. However, because users freely tag images with labels, these user-assigned labels are often ambiguous. The label "albatross", for example, may refer to a type of bird or aircraft. If the ambiguities are resolved, we can use Wikipedia entries for cataloging as an alternative to ontologies. To formalize this, we propose a task called *image label disambiguation* where, given an image and assigned target labels to be disambiguated, an appropriate Wikipedia page is selected for the given labels. We propose a hybrid approach for this task that makes use of both user tags as textual information and features of images generated through image recognition. To evaluate the proposed task, we develop a freely available test collection containing 450 images and 2,280 ambiguous labels. The proposed method outperformed prevalent text-based approaches in terms of the mean reciprocal rank, attaining a value of over 0.6 on both our collection and the ImageCLEF collection.

Keywords: Entity Linking, Multimedia, Wikipedia, Test Collection.

1 Introduction

In recent years, large amounts of analog materials have been digitized by libraries and museums to preserve their contents [1]. This has led to a large amount of multimedia collections consisting of image and text data. When these data are archived, librarians assign appropriate metadata to them to catalogue them for reference. Manual annotations for these ever-growing digital archives are expensive. To solve this problem, we propose a mapping system that links image and textual multimedia data to appropriate

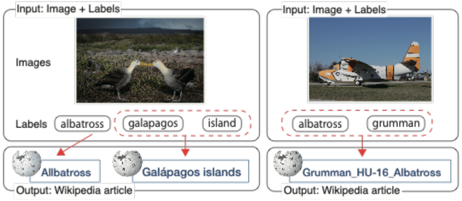

Fig. 1. "albatross" label is linked to albatross (bird) Wikipedia page on the left. By contrast, "albatross" label along with "grumman" label are linked to an aircraft model page on the right.

entries on Wikipedia as a replacement for cataloging. The use of the web data, especially Linked Data, to supplement ontologies for cataloging has been attempted for music archives [2] and has been discussed in the context of libraries [3, 4]. Our mapping utilizes Wikipedia for an ontology of collections and processes of annotating metadata to these collections. It can thus be beneficial in various ways, not only for automatic annotations but also for accommodating emergent metadata quickly updated by users on Wikipedia as an ontology and for implementing Q&A systems. This is an example of the application of such a system that can answer visitors' questions about the contents of exhibited images using knowledge from Wikipedia.

We first target images and the associated labels linked from the photo-sharing site Flickr[1] to articles on Wikipedia. When users upload photos to the site, they tag them with labels describing the photos [5]. However, users tend to freely assign labels and ambiguities naturally occur in the designation. If these ambiguities are solved for, we can link Wikipedia entries with records of image collections as catalogue metadata.

To formalize the task, we introduce the task *image label disambiguation (ILD)*, in which an image and its associated user-assigned labels are provided as a query, the system in response identifies an entry represented by labels in Wikipedia. **Fig. 1** illustrates the overview of the task. The two pictures shown are examples from Flickr in which the same label, "albatross", is used to refer to different types of entities. The image on the left is that of the bird called albatross whereas the one on the right is that of an aircraft of the same name. In the ILD task, the system links the label "albatross" either to the bird or the aircraft model.

There are two general ways of performing the above task. One involves using other labels (e.g., the label "Galápagos islands" provides a hint that the image on the left may be that of the bird native to the Galápagos Islands) or image features (e.g., the image on the right has a shape more similar to that of an aircraft than that of a bird).

Labels are used as text data in traditional entity linking. The entity linking task has been extensively studied in the context of selecting a Wikipedia page to which to link a given mention of an entity in text (the so-called "wikification" task) [6, 7, 8, 9]. In general, this line of work has focused on linking entity mentions found in narrative text,

[1] https://www.flickr.com/

e.g., newspaper articles, whereas image descriptions, which is our focus in the ILD, often contain a few words.

The other ways of disambiguating labels of images involves image recognition. Image recognition using deep learning has been actively studied in the last decade. Although the performance of recent classifiers has been impressive, the applicability of image classifiers to ILD is restricted because of the scarcity of training data. Image classification requires large amounts of training data. Preparing enormous amounts of data covering all possible entities, such as the "Grumman HU-16 Albatross" aircraft model, is unrealistic.

There are shortcomings in both the above approaches when applied to ILD. We propose solving ILD by combining textual and image features to remedy the defects of each. Classification labels, such as "albatross (bird)" for the image on the left and "aircraft" for that on the right in **Fig. 1**, can be used as clues for disambiguation. Based on this idea, we propose a hybrid approach using user labels and image features generated by CNN classifiers.

Since ILD is a new task that requires a testbed, we develop a test collection by combining both of the aspects of wikification with image classification. The collection consisted of 450 images and a total of 2,280 ambiguous target labels. Compared with the test collection for image classification, this collection was small because it was manually annotated. However, our initial focus is on establishing a highly accurate linking approach for this collection as a first step in research on ILD.

The contributions of this paper are threefold:

1. We introduce a task called image label disambiguation in which, given an image and associated user labels as input, the system links the labels to Wikipedia pages for the corresponding entity.
2. We propose a hybrid approach that uses labels of images and objects generated by image classifiers using a CNN. The results of experiments show the superior retrieval performance of the proposed approach to prevalent approaches, with an improvement of approximately 0.1 MRR on two test collections.
3. We develop a freely available test collection[2] containing 450 images and 2,280 ambiguous target labels for evaluating ILD systems.

The remainder of this paper is structured as follows: Section 2 summarizes related work and Section 3 introduces test collections used in the evaluation and details of the development of our ILD test collection. Section 4 describes our proposed approach, Section 5 describes experiments used to evaluate it and Section 6 concludes this paper.

2 Related Work

In this section, we summarize research in three areas: image recognition, entity linking, and test collection.

[2] https://zenodo.org/record/3353813

Image recognition. Image recognition using neural network models has been actively studied in the last decade [10, 11, 12, 13]. Neural network architectures of image recognizers now use neural network models with deep layers of convolutional networks, such as in AlexNet [10] and VGGNet [11]. These structures have exhibited impressive performance on image recognition tasks. Recent models [12, 13] have explored deeper layers in these architectures. However, applying image recognition to ILD may not be suitable due to the difficulty of preparing training data, which is crucial for adequate classification performance in image recognition. For example, the concept of the Galápagos Islands in **Fig. 1** has too many visual representations for the classification. Furthermore, image recognition using neural network models requires large amounts of training resources. Preparing massive amounts of training data with labels over specific entities is unrealistic.

Entity linking in text or image. Entity linking is the task of identifying entity mentions in text passages and linking them to corresponding entities in a knowledge base, especially called wikification in the case of Wikipedia. Wikification systems have been extensively studied. Cucerzan's approach, for example, uses similarity based on Wikipedia anchor text (words that label a web link) [6], whereas some studies make use of graphs. Ratinov *et al.* leveraged the link structure of Wikipedia as a basis for disambiguation [7]. Moro proposed a graph-based approach for the sematic interpretation of text. Cheng and Roth, by contrast, leveraged linguistic features, coreference relations and named entity recognition [8]. Ganea used contexts with a probabilistic model [9]. In general, this line of work has focused on linking entity mentions in narrative text (e.g., newspapers articles), whereas image labels, which are our focus in ILD, often contain few words.

Entity linking has also been studied for images and is called *visual entity linking* [14, 15]. It involves detecting regions of objects in images and linking them with entities in the associated description. In one example in a past paper, a motorcyclist and bike were identified from the target description in Flickr8k [16] "A motorcycle racer leans his bike". But we want more granular entities, such as the model of the motorcycle that users specify for the image. Moreover, in contrast to visual entity linking, all user-assigned labels do not have corresponding regions in images, e.g., Galápagos islands for the left of **Fig. 1**.

Test collection. Test collections for images have been developed for image recognition tasks such as the PASCAL VOC [17], MS COCO [18] and ILSVRC [19]. However, the classification systems of these collections are too broad for the assessment of ILD. The PASCAL VOC contains 20 classes of notional taxonomy and MS COCO contains 92 common object categories. The ILSVRC 1,000 synset task collection uses ImageNet [20] ontology for classification. However, these image recognition collections have no user-assigned labels. For image annotation tasks, NUS-WIDE [21] contains Flickr images with user-assigned labels. The correspondence of the labels and their entities have not been solved for, however.

3 Test collection

In this section, we describe two test collections that can be used for ILD evaluation. First, we briefly explain the ImageCLEF collection [22] for the scalable image annotation task. Second, we describe the development of a new test collection (Animal Name collection).

3.1 ImageCLEF collection

The ImageCLEF collection was developed for the scalable image annotation task where systems automatically annotate input pictures collected from the web. The annotations were based on popular user queries for image searches.

This collection consists of 7,291 images with 207 manually annotated concepts. The concepts are defined as in WordNet [23] synsets and have links to Wikipedia articles in most cases. This correspondence between the concepts of images and Wikipedia entries allowed us to evaluate ILD systems on the ImageCLEF collection, for which we selected 53 of the concepts that had associated Wikipedia pages and had been defined in the 1,000 classes of ILSVRC [19] as target entities for disambiguation. The latter condition was used to evaluate approaches relying on the 1,000-class classifier (explained in Section 4). The 53 entities featured a variety of types, such as guitars, mushrooms and lakes (the list of the selected entities is available in our uploaded test collection). Using these entities, we selected images assigned to them in the concepts. As a result, 1,197 images and the assigned concepts were available for evaluation.

3.2 Building a test collection for ILD

ImageCLEF can be used for ILD evaluation. However, the concepts of the collection are general words reflecting searchers' queries for images. We are interested in more granular target entity types, such as the Grumman model HU-16 Albatross shown in **Fig. 1**. This motivates us to develop a test collection designed for ILD evaluation. To formulate the gold standard of correspondences between labels of images and granular entity types, we annotated the entries in the new collection.

Data collection. We first defined categories of the test collection. We used the policy whereby labels of the test collection of ILD can map to several entities leading to ambiguity. We chose animal names because they are often used as brand names of products or nicknames of sports teams, and this satisfies the ambiguity requirement. We selected 15 animal names listed on a Wikipedia page[3] of 512 general animal names.

We then collected images and tagged labels assigned by users on Flickr by searching the chosen animal names as queries. We randomly selected 30 images for each animal name as labels. The selected images were not limited to pictures of animals, but included any genre, e.g., automobile and airplane. The label "jaguar", for example, refers

[3] https://en.wikipedia.org/wiki/List_of_animal_names

not only to a wild cat but also a luxury car brand. **Fig. 2** shows the distribution of the main subjects of the pictures. It represents the ambiguity of the selected names, which is desirable for the evaluation. The average number of labels for each category was 180 after camera specification labels had been eliminated, e.g., “nikond5” and “500mmf4”.

Table 1. Examples of Entities in two test collections

Test Collection	Examples of entities
Animal Name collection	Albatross (bird), Wandering albatross, Grumman HU-16 Albatross, Cricket (sports), Cricket (insect), Jaguar Cars, Jaguar
ImageCLEF 2014 collection	Banana, Bicycle, Cauliflower, Cheetah, Drum, Lion, Potato

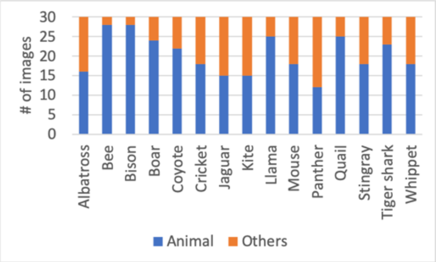

Fig. 2. Distribution of subjects of pictures

Annotation. We recruited five annotators who were graduate students and had studied English as a second language. Each of them was assigned six categories to annotate that were balanced to nearly the same volume across annotators. Two annotators were assigned to each category.

The annotators subjectively determined entities to link to labels of an image. An image and its labels were given to the annotators on a spreadsheet. They identified a label (or labels) which should refer to a given entity in the English version of Wikipedia regardless of whether it was depicted in the picture. Using the labels “Galapagos” and “island” in **Fig. 1** as an example, the annotators were asked to judge whether the labels were linked to the Galápagos Islands from the image and the other labels. They then searched Wikipedia pages for entities of the labels by querying their single or multiple labels. In this step, they could modify their queried labels within their interactions with the search results of Wikipedia to obtain candidates. Finally, they judged whether the retrieved pages were appropriate for the label and, if so, assigned the Wikipedia pages as the correct entity on their spreadsheets. If they determined that the labels did not correspond to any Wikipedia page, they assigned a NIL entity to the relevant labels. After three trials of the above procedures, they began annotation. We examined the quality of the outcomes of annotation by investigating inter-annotator agreement and Cohen’s κ coefficient between them [24]. Their agreement was indicated with a value of 0.8, and when κ was over 0.9, this implied almost perfect agreement.

Finally, we created a test collection of 2,280 ambiguous target labels of 450 images, which showed high inter-annotator agreements. The number of images was not as large as that of image test collections intended for supervised learning. The number of labels, however, was large for the testbed, three times that of test collections with a maximum of 700 mentions used for the wikification of text [8]. **Table 1** compares examples of entities in the collection of Animal Name and the ImageCLEF collection. Our collection covered ambiguities among entities, which is desirable for ILD evaluations.

4 An Approach with Use of Textual Labels and Object Recognition

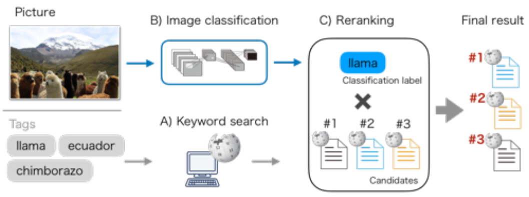

Fig. 3. Overview of proposed approach

This section describes our proposed approach for disambiguating user labels. The approach is generally a reranking method based on the results of a keyword search system. An overview is provided in **Fig. 3**. It consists of three steps: A) keyword search system, B) image classification and C) reranking.

A) The purpose of the keyword search step is to obtain candidate Wikipedia pages for image labels. To generate the pages, we employed a keyword search system with BM25 similarity scoring implemented in Apache Solr (ver. 7.3.0)[4]. All pages for each image label in the search results were used as candidate pages while C) reranking.

B) In the image classification step, we extract the objective information of an input image by using deep learning image classifiers. We implemented two types of classifiers with different policies to generate objective information. B-1) The first classifier was trained on the primal collection categories described in Section 3. It classified an input image to one of these categories. Because collection categories can be a subject in a picture, our aim in using this classifier is to obtain the collection category as an objective label with high accuracy. B-2) The second classifier aimed to obtain as much objective information as possible. The classified labels were not limited to primal collection categories and included other objects in a picture. For example, **Fig. 3** shows the picture of the collection category "llama". If another label "alp" is obtained by the classifiers, it can be useful to infer the mountain-related contents of the image and disambiguate it from the label "chimborazo" (an inactive volcano in the Andes). Because images may contain several objects, we used the top five classification labels per image.

B-1) The aim of classifying categories of collection (Category classifier) is to obtain highly accurate primal categories of the input pictures. To this end, we utilize a recent convolutional architecture called the *deep residual network (ResNet)* [12]. In this network, each layer is connected through a direct connection and an additional connection of the input layer that formulate the layer as a residual learning function. Using this framework, residual networks can train substantially deeper models that deliver better performances.

[4] http://lucene.apache.org/solr/

The training data were collected by downloading images from ImageNet[5], where these images were assigned to classes of ImageNet corresponding to the collection categories. We had 400 and 650 images, determined by the minimum number of collected images in each category, in the Animal Name and ImageCLEF collections, respectively. We splitted up the collected images into 70% for training and 30% for validation. We used a 152-layer model of ResNet[6] trained on 1,000 object classes of ImageNet with the replacement of the last fully connected layer with the output layer for the collection categories, and trained the model with a mini-batch size of 32 and an SGD optimizer. The initial learning rate, weight decay and the momentum were 10^{-4}, 10^{-6} and 0.9, respectively. The accuracy of the trained classifiers was 0.65 on the Animal Name collection and 0.66 on ImageCLEF. The output labels and the probability (confidence score) of the *softmax* layer were used in the C) reranking step.

B-2) We used a multi-label classifier trained on 1,000 object classes of the ILSVRC classification task [19] (1k classifier). A total of 1,000 synsets were selected from ImageNet so that there was no overlap between them; any given synset was not an ancestor of the others in the ImageNet hierarchy. This is an advantage of using the category set. The dataset category contained a variety of classes, such as animals, locations, sports activities, stationery items and gadgets, which are beneficial for inferring the contexts of the pictures. To implement this classifier, we used a pre-trained classifier[7] using Keras's implementation of the ResNet [12] model trained on over 1.2 million images.

C) In this step, we used the similarity between candidate Wikipedia pages from A) and a pair of image labels originally tagged by users and objective labels from B). We used Wikipedia pages from only A) and discarded the ranking and score of the search results to measure the relation between the Wikipedia pages and the labels.

Because the vocabulary of the classification labels was limited to the trained class system, term-matching retrievals could not distinguish among the relatedness of the labels to entities if they were in different vocabularies. To handle the relatedness of words in the ranking, we employed word embeddings using *word2vec* [25] to this similarity calculation, motivated by its recent success in such ranking tasks as web search [26] and document retrieval in software engineering [27]. Word2vec learns word embeddings by maximizing the log-conditional probability of a word given words within a fixed-sized window, so that words with similar meanings are associated with similar vectors. Word vectors, which contain useful knowledge about the distributional properties of words, allow the ranking to recognize relations. For example, "alp" was a classification labels of B-2 for the image in **Fig. 3**, which indicates mountain-relatedness and closeness to "chimborazo", an inactive volcano in South America, although term-matching retrievals overlooked similarity.

[5] https://www.image-net.org/
[6] https://keras.io/applications/#resnet

There are two models for learning word embeddings in word2vec: *skip-gram* and *continuous bag-of-words*. Both models produce similar embeddings in terms of quality and quantity. We used the skip-gram model with a window size of five to learn word vectors of 300 dimensions using English Wikipedia dump data on 1st October 2017.

The overall similarity was computed by the similarity between the Wikipedia pages and pairs of user labels, w_q and objective labels, w_l defined as follows:

$$\mathrm{sim}(T, Q) = \alpha \sum_{w_q \in Q} \mathrm{sim}(T, w_q) + (1 - \alpha) \sum_{w_l \in L} \mathrm{sim}(T, w_l), \tag{1}$$

where T represents the titles of candidate Wikipedia pages, Q represents the set of user labels of images and L represents a label or labels automatically generated by image classifiers. In preliminary experiments, using words in the titles of articles to compute similarity among them delivered the best retrieval performance after several trials of document modeling. α in the above represents a weighting parameter of similarities on labels and objective labels. In computing the similarity of automatic labels, similarities to Wikipedia pages were weighted by using the confidence scores of the classifiers:

$$\mathrm{sim}(T, w_l) = \frac{1}{|T|} \sum_{w_t \in T} \mathrm{sim}(w_t, w_l) \times \mathrm{score}(w_l), \tag{2}$$

where w_t is a word in a Wikipedia document, w_l is a word in classification labels and $\mathrm{score}(w_l)$ is a confidence score of the prediction of the given label by the classifiers. Similarity between words was computed by the cosine distance of their vector representations:

$$\mathrm{sim}(w_1, w_2) = \cos(\mathbb{w}_1, \mathbb{w}_2) = \frac{\mathbb{w}_1^T \mathbb{w}_2}{\|\mathbb{w}_1\| \|\mathbb{w}_2\|}. \tag{3}$$

This is the inner product of two vectors $\mathbb{w}$ of words w learned by word embedding. Although *out-of-vocabulary (OOV)* words also pose challenges to similarity computation, as mentioned by Mitra [26], we ignored OOV words here.

5 Experiment

The experiment aimed to answer following two questions: First, to what extent can the proposed approach solve ILD tasks compared with other approaches in the literature? For this, we evaluated several approaches on image label disambiguation targeting all given labels of images. Following this, when labels that represented objects in images were only used, this was considered image classification. The question was that of whether the proposed approach, which uses both text labels and image features, could outperform image classifiers that use only image features. For this, we evaluated several approaches on image classification tasks.

Two test collections, as explained in Section 3, were employed in this evaluation. One is our Animal Name collection containing 2,280 ambiguous labels of 450 images. The other was the ImageCLEF collection. We used 1,197 images using 53 concepts.

The disambiguation targets were 10,573 conceptual labels in total. For the entity set, English Wikipedia dump data on 1st October 2017, which contained 5,486,204 pages, were used.

Table 2. Image label disambiguation results. ** means statistical significance compared to results of BM25 at 0.01 level.

	Animal Name collection			Image CLEF 2014 collection		
Method	MRR	R@1	R@10	MRR	R@1	R@10
TF-IDF	0.480	0.436	0.532	0.668	0.632	0.706
BM25	0.509	0.471	0.577	0.627	0.595	0.706
Wikification	0.523	0.481	0.601	0.628	0.595	0.708
Method 1	**0.609	0.558	0.684	**0.715	0.711	0.718
Method 2	**0.583	0.518	0.679	**0.719	0.711	0.730

5.1 Image Label Disambiguation Setting

In this setting, given image labels as a query, the system returned a ranked list of Wikipedia pages for the relevant entities. In this task, all image labels were targets to be disambiguated and used as queries. For example, five labels—“albatross”, “galapagos” and “island” on the left in **Fig. 1**, and “albatross” and “grumman” on the right—were used for image label disambiguation. The number of labels in each query, i.e., single or multiple labels, was determined in the annotations according to Section 3.

We evaluated five approaches: TF-IDF, BM25, Wikification, Method 1 and 2. Method 1 and 2 are our proposed approaches. Method 1 used a collection category classifier and Method 2 the 1k classifier (B-1 and B-2 in Section 4, respectively). TF-IDF and BM25 are text retrieval systems. Retrieving English Wikipedia by querying using user labels does not much differ from traditional retrieval, so that we used $k_1 = 1.2, b = 0.75$ in Okapi at TREC-3 as free parameters settings for BM25 [28].

The wikification system (Wikification) was proposed by Cucerzan [6]. Considering that user labels tagged to a picture consisted of a few keywords, where the linguistic structure was rarely maintained, this method is a better choice than other wikification systems that rely on the linguistic features mentioned in Section 2. Wikification retrieves correct entities for a query by computing cosine similarity between word occurrence vectors of an input document and the candidate Wikipedia pages with same surface forms as that of the use of the label. We prepared all pages listed in a Wikipedia disambiguation page for a queried label as candidate Wikipedia pages.

Table 2 shows the results in terms of MRR [29] and Recall@1, 10. The proposed approaches achieved the best performance in both collections. The MRR of Method 1 was 0.609 on the Animal Name collection and that of Method 2 was 0.715 on ImageCLEF. These results were superior to those of BM25 used in reranking steps in Method 1 and 2. We also assessed statistical significance between the results of BM25, and Method 1 and 2 with a two-tailed paired t-test. The difference was significance at a 0.01 level.

Some successful cases of Method 1 and 2 are shown in **Fig. 4**. The figure displays pairs of an image and the correct entity, where Method 1 or 2 output the correct entity but the other approaches could not. Our approach, for example, generated both a coyote and a Coyote Buttes for the label "coyote". On this category, Method 1 achieved a higher improvement in MRR than BM25. The major errors, by contrast, appeared in users' free-written labels, which are different from the entity in their surfaces. Neither method could generate the correct entity on its list for 20% of the labels in the Animal Name collection.

Wikification and TF-IDF yielded the second-highest values of MRR. The two tended to output a particular page with high rank in their results for same labels regardless of the visuals of the image. For example, when the query label was the "jaguar" label of an animal or pictures of cars, they persistently ranked a wild cat jaguar page at the first and the page for the carmaker Jaguar around the 50th in their ranked lists.

Analysis of results. We investigated the influence of the weighting parameter α, representing similarities between user labels and classification labels in Equation (1), on MRR. **Fig. 5** shows the MRR of Method 1 and 2 for a value of α of 0.1. The similarity in user labels highly influenced the MRR in both collections. In particular, the MRR of ImageCLEF was flat at any given α, possibly because ImageCLEF labels of general search queries were not ambiguous. On the animal collection, by contrast, MRR increased with α, which also indicates the dominance of user labels in the similarity computation. However, because we fixed α for all categories of the collection to evaluate its influence, the maximum MRR in **Fig. 5** was 0.52, smaller than that at the best setting of α in **Table 2**. Therefore, image features can improve the disambiguation of labels.

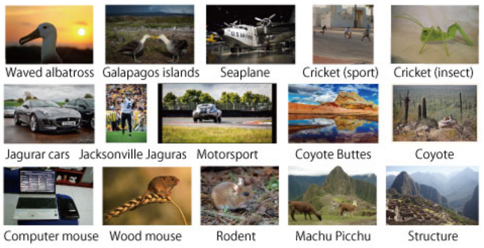

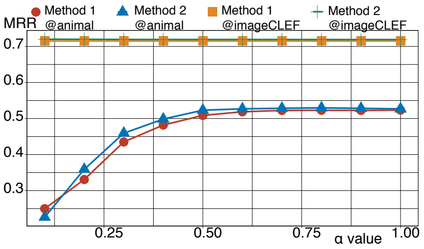

Fig. 4. (Left) Successful pairs of images and entities by Method 1 and 2.

Fig. 5. (Right) MRR at each value of a parameter.

5.2 Image Classification Setting

We picked images and the labels of objects. For the ImageCLEF collection, we used all 1,197 images and our 53 categories. For the Animal Name test collection, we selected images that had been assigned object labels referring to animals. Taking **Fig. 1** as an example, we used the label "albatross" for the left image but did not use it for the image on the right because the correct referent of "albatross" was linked to an aircraft entity that is not in animal. In total, 277 images with 15 animal categories were used.

We compared Method 1 and 2 with the two image classifiers B-1 and B-2 in Section 4. The first classifier was trained on 1,000 classes (1k classes) of ILSVRC and the

second on categories of the two collections (Category).

We evaluated the accuracy of classification as $\text{Accuracy} = \text{\# of correctly classified images} / \text{\# of images in category}$, where we considered an image to have been correctly classified when the correct object label was listed in the top five predictions of the 1k classes and Category classifiers because the pictures are tagged with several labels—fewer than five labels in most cases. For Method 1 and 2, the results were counted as correct when they generated the correct Wikipedia page in the first rank in the retrieval results for a queried label.

Table 3 shows the average accuracy of the examined approaches. The proposed approaches achieved the best classification at 0.958 on the Animal Name and one on the ImageCLEF collections. Method 1 and 2 delivered better performance than the 1k classifier and the category classifier, which indicates that they can make use of user labels as text information for classification. A notable case of this success is a spray-painted image of a bee in the bee category, where Method 1 succeeded while the others failed. Although the proposed method generally showed high accuracy, that of Method 2 was low at 0.789 on the Animal Name collection. This was because of failure in the reranking step, even though the correct classification labels were generated by the 1k classifier.

While category had high accuracy above 0.7, 1k classes exhibited low accuracy, primarily because of the differences in the collection of images between Flickr and ImageCLEF, and ImageNet, on which the 1k classes' model was trained. Compared with ImageNet, which arranged one class per image, image in Flickr and ImageCLEF were from the web, and usually included multiple categorial images, often three or more. The 1k classifier failed on these images.

Table 3. Image classification results (average accuracy)

Test Collection	1k classes	Category	Method 1	Method 2
Animal Name collection	0.803	0.889	0.958	0.789
ImageCLEF 2014 collection	0.680	0.741	0.995	1

6 Conclusion

In this paper, we proposed a task called image label disambiguation (ILD), in which the system retrieves correct entities in response to queried ambiguous labels associated with an image, with the aim of automatically cataloging multimedia collections. To evaluate ILD systems, an ILD test collection was developed that is freely available to users and developers. We developed an approach to disambiguate labels by employing user labels of images and a CNN classifier for image classification.

In future work, more sophisticated document modeling, such as using the average of word vectors in a document, will be considered. With regard to ILD approaches, we plan to test a combination of two types of classifiers to further improve the results. We also plan to increase the size of the test collection for more robust evaluation.

References

1. Corrado, E. M.: *Digital Preservation for Libraries*, Archives, and Museums (2nd ed.). Rowman & Littlefield Publishers, Cambridge, MD, USA. (2017).
2. Page, K. R., Bechhofer, S., Fazekas, G., Weigl, D. M. and Wilmering, T.: Realizing a Layered Digital library: Exploration and Analysis of the Live Music Archive through Linked Data. In: *Proceedings of the 17th ACM/IEEE Joint Conference on Digital Libraries*, pp. 89–98. (2017).
3. Kroegen, A.: The road to BBIBFRAME: the evolution of the idea of bibliographic transition into a post-MARC Future. *Cataloging & Classification Quarterly* 51(8), 873–890. (2013).
4. Nurmikko-Fuller, T., Jett, J., Cole, T., Maden, C. Page, K. R. and Downie, J. S.: A Comparative Analysis of Bibliographic Ontologies: Implications for Digital Humanities. In: *Digital Humanities 2016: Conference Abstracts*, pp. 639–642. (2016).
5. Sawant, N., Li, J. and Wang, J. Z.: Automatic image semantic interpretation using social action and tagging data. *Multimedia Tools and Applications*. 51(1), 213–246. (2011).
6. Cucerzan, S., Large-Scale Named Entity Disambiguation Based on Wikipedia Data. In: *Proceedings of the 2007 Joint Conference on Emprical Methods in Natural Language Processing and Computational Natural Language Learning*, pp. 708–716. (2007).
7. Ratinov, L., Roth, D., Downey, D. and Anderson, M.: Local and Global Algorithms for Disambiguation to Wikipedia. In: *Proceedings of the 49th Annual Meeting of the Association for Computational Linguistics: Human Language Technologies*, pp. 1375–1384. (2011).
8. Cheng, X. and Roth, D.: Relational Inference for Wikification. In: *Proceedings of the 2013 Conference on Empirical Methods in Natural Language Processing*, pp. 1787–1796. (2013).
9. Ganea, O. E., Ganea, M., Lucchi, A., Eickhoff, C. and Hofmann, T.: Probabilistic Bag-Of-Hyperlinks Model for Entity Linking. In: *Proceedings of the 25th International Conference on World Wide Web*, pp. 927–938. (2016).
10. Krizhevsky, A., Sutskever, I. and Hinton, G. E.: mageNet Classification with Deep Convolutional Neural Networks. In: *Proceedings of the 25th International Conference on Neural Information Processing Systems*, pp. 1097–1105. (2012).
11. Simonyan, K. and Zisserman, A.: Very Deep Convolutional Networks for Large-Scale Image Recognition. *arXiv preprint arXiv:1409.1556*. (2014).
12. He, K., Zhang, X., Ren, S. and Sun, J.: Deep Residual Learning for Image Recognition. In: *Proceedings of the 29th IEEE Conference on Computer Vision and Pattern Recognition*, pp. 770–778. (2016).
13. Szegedy, C., Loffe, S., Vanhoucke, V. and Alemi, A. A.: Inception-v4, Inception-ResNet and the Impact of Residual Connections on Learning. In: *Proceedings of the 31st AAAI Conference on Artificial Intelligence*, pp. 4278–4284. (2015).
14. Weegar, R., Hammarlund, L., Tegen, A., Oskarsson, M., Åström, K. and Nugues, P.: Visual Entity Linking: A Preliminary Study. In: *Proceedings of Cognitive Computing for Augmented Human Intelligence workshop at the 28th AAAI Conference on Artificial Intelligence*. pp. 46–49. (2014).
15. Tilak, N., Gandhi, S. and Oates, T:. Visual entity linking. In: *Proceedings of the 2017 International Joint Conference on Neural Networks*. pp. 665–672. (2017).
16. Hodosh, M., Young, P. and Hockenmaier, J.: Framing image description as a ranking task: data, models and evaluation metrics. *Journal of Artifical Intelligence Research*. 47(1), 853–899. (2013).

17. Everingham, M., Eslami, S. M. A., Gool, L. V., Williams, C. K. I., Winn J. and Zisserman A.: The Pascal Visual Object Classes Challenge: A Retrospective. *International Journal of Computer Vision*, 111(1), 98–136. (2014).
18. Lin, T. Y., Maire, M., Belongie, S., Bourdev, L., Girshick, R., Hays, J., Perona, P., Ramanan, D., Zitnick, C. L. and Dollár, P.: Microsoft COCO: Common Objects in Context. In: *Proceedings of the 13th European Conference on Computer Vision*, pp. 740–755. (2014).
19. Russakovsky, O., Deng, J., Su, H., Krause, J., Satheesh, S., Ma, S., Huang, Z., Karpathy, A., Khosla, A., Bernstein, M., Berg, A. C. and Fei-Fei, L.: Imagenet large scale visual recognition challenge. *International Journal of Computer Vision*, 115(3), 211–252. (2015).
20. Deng, J., Dong, W., Socher, R., Li, L., Li, K. and Fei-Fei L.: ImageNet: A large-scale hierarchical image database. In: *Proceedings of the 2009 IEEE Confernce on Computer Vision and Pattern Recognition*, pp. 248–255. (2009).
21. Chua, T. S., Tang, J., Hong, R., Li, H., Luo, Z. and Zheng, Y.: NUS-WIDE: A Real-World Web Image Database from National University of Singapore, In: *Proceedings of the 8th ACM International Conference on Image and Video Retrieval*. (2009).
22. Gilbert, A., Piras, L., Wang, J., Yan, F., Ramisa, A., Dellandrea, E., Gaizauskas, R. and Mikolajczyk, K.: Overview of the ImageCLEF 2016 Scalable Web Image Annotation Task. In: *Proceedings of the Seventh International Conference of the CLEF Association*. (2016).
23. Fellbaum, C.: *WordNet: An Electronic Lexical Database*. MIT Press, Cambridge, MA, USA. (1998).
24. Carletta, J.: Assessing agreement on classification tasks: the kappa statistic. *Computational Linguistics*. 22(2), 249–254. (1996).
25. Mikolov, T., Sutskever, I., Chen, K., Corrado G. and Dean, J.: Distributed Representations of Words and Phrases and their Compositionality. In: *Proceedings of the 26th International Conference on Neural Information Processing Systems*, pp. 3111–3119. (2013).
26. Mitra, B., Nalisnick, E., Craswell, N. and Caruana, R.: A Dual Embedding Space Model for Document Ranking. *arXiv preprint arXiv:1602.01137*. (2016).
27. Ye, X., Shen, H., Ma, X., Bunescu, R. and Liu, C.: From Word Embeddings To Document Similarities for Improved Information Retrieval in Software Engineering. In: *Proceedings of the 38th IEEE International Conference on Software Engineering*, pp. 404–415. (2016).
28. Robertson, S. E., Walker, S., Jones S., Hancock-Beaulieu, M. M., and Gatford M.: Okapi at TREC-3. In: *Proceedings of the third Text REtrieval Conference*, pp. 109–126. (1996).
29. Craswell, N.: Mean Reciprocal Rank, In: Liu, L. and Özsu, M. T. (Eds.), *Encyclopedia of database systems*. Springer, Boston, MA, USA. (2009).
